# Medical Mistrust on Prostate Cancer Screening: A mixed method study among African Americans, Caribbean immigrants and African immigrants

**DOI:** 10.18103/mra.v12i8.5727

**Published:** 2024-08-31

**Authors:** Malika Nipher, Roberts Lisa, Alemi Qais, Casiano Carlos A, Montgomery Susanne

**Affiliations:** a RAND Corporation, 1776 Main St., Santa Monica, CA 90401, USA; b Loma Linda University School of Nursing, 11262 Campus Street, West Hall, Loma Linda, CA 92350 USA; c Loma Linda University, School of Behavioral Health, 11065 Campus St., Loma Linda, CA 92350 USA; d Center for Health Disparities and Molecular Medicine, Loma Linda University School of Medicine, 11085 Campus Street, Mortensen Hall, Loma Linda, CA 92350 USA

**Keywords:** Prostate Cancer, medical mistrust, African America, African immigrant, Caribbean immigrant, screening

## Abstract

**Objectives:**

The contribution of medical mistrust to healthcare utilization delays has been gaining increasing attention. However, few studies have examined these associations among subgroups of Black men (African Americans, Caribbean, and African immigrants) in relation to prostate cancer (PCa). This study addresses this gap by assessing how medical mistrust affects PCa screening behavior and to further understand perceptions of medical mistrust among subgroups of Black men.

**Methods:**

This research employs a mixed-methods approach comprising two distinct phases. In Phase 1, a cross-sectional examination was conducted to evaluate the influence of medical mistrust toward healthcare organizations on prostate cancer screening among 498 Black men. In Phase 2, a qualitative investigation was undertaken to delve into the nuances of medical mistrust through six focus groups (n=51) and ten key informant interviews (n=10). Logistic regression and grounded theory methods were employed for data analysis.

**Results:**

Quantitative findings unveiled disparities in mistrust among subgroups, with Caribbean immigrants exhibiting higher levels of medical mistrust. Nevertheless, individuals with a family history of PCa showed elevated likelihoods of undergoing screening, despite mistrust. Qualitative results revealed 1) differences in reasons for medical mistrust among Black subgroups, 2) cultural perceptions which influence medical mistrust and medical care seeking, 3) lack of education in relation to PCa that contributes to medical mistrust, 4) negative past experiences and poor provider communication contribute, and 5) when PCa directly affected one’s life, either personally or within the family, there was a recognized importance placed on monitoring one’s risk despite mistrust.

**Conclusion:**

While medical mistrust may not significantly deter healthcare utilization among individuals with a family history or diagnosis of PCa, it underscores the variability of medical mistrust and its underlying reasons among different Black subgroups.

## Background

Despite growing efforts to eliminate racial and ethnic health inequities, Black men’s life expectancy at birth in the United States (US) is among the shortest compared to other racial/ethnic groups.^1, 2^ Studies have shown that this shortened life-expectancy is a result of the interplay between economic, biological and socio-structural factors.^1, 3^ Such factors include high disease morbidity and mortality rates, well documented experiences of structural racism, and diminished access to opportunities for upward social mobility.^4–7^ Moreover, men are less likely than women to use preventive health services^8, 9^ and Black men have the lowest engagement in timely preventative health screenings and medical treatment.^10, 11^ Reasons for this include psychosocial reasons such as limited health knowledge,^10, 12^ fatalism,^13^ socioeconomic barriers,^13, 14^ masculinity beliefs,^8, 15, 16^ perceived racism,^10, 17^ and medical mistrust.^8, 18^

In addition to the historically documented horrific experiences with systemic abuse such as the Tuskegee health study,^19^ medical mistrust is increasingly recognized as a social determinants of health. This mistrust within medical systems and personnel, perceived to represent the dominant culture in society, contributes significantly to why underserved and discriminated groups in the US tend to participate less in preventative measures and healthcare, often seeking asstance only when it’s too late.^20–24^. Medical mistrust is associated with lower health care satisfaction,^25^ lower health care utilization,^23^ and it negatively affects preventive health practices.^23, 26^ Researchers argue that medical mistrust is group-based, stemming from the tendency to distrust systems where one’s group is poorly represented.^22, 27^ This distinction from interpersonal trust is crucial; medical mistrust doesn’t signify ‘no trust’, but rather implies the belief that the trustee act against one’s best interest.^28^ Among African Americans, medical mistrust is reported to be the highest of any other racial or minority group.^29^

Prostate cancer (PCa) disproportionately affects Black men in the US.^30, 31^ They tend to develop PCa at younger ages compared to other racial/ethnic groups, often present with a more aggressive form of PCa, and die at a higher rate than any other groups.^31, 32^ Additionally, Black men are less likely to undergo screening and treatment,^32–34^ which for PCa involves prostate-specific antigen (PSA) testing and digital rectal examination (DRE). To complicate this issue further, PCa screening guidelines are confusing, not only to men but also to providers.^33, 35^ While PCa guidelines for most other groups call for watchful waiting and have moved away from PCa screening, guidelines for Black men have gone through a variety of changes over time as their disease presentation is so different.^36, 37^ For example, given cost/benefit concerns the US Preventive Services Task Force (USPSTF) originally recommended against PSA screening for men of any age in 2012,^36^ despite the underrepresentation of Black men in the screening trials that formed the basis for this recommendation.^38^ On the other hand, the American Cancer Society, the American Urologic Association, and the National Comprehensive Cancer Network continued to recommend individualized screening decisions, especially for Black men at elevated risk.^39–41^ Given the PCa disparity for Black men, experts suggest more frequent and early PSA and DRE screening to mitigate survival disparities.^42^ Unfortunately, the 2012 USPSTF guideline change led to a decline in screening among Black men, who already had low screening rates, resulting in increased deaths.^43^ In response to the backlash prompted by this change, the USPSTF reversed their recommendations for Black men.^33, 37^ However, many physicians have remained unaware of this reversal, and the ongoing debates around testing have further eroded trust among those most affected by PCa. The latest recommendation from the USPSTF, issued in 2018, applies to men aged 55 to 69 and emphasizes that the choice to undergo periodic PSA-based screening for PCa should be made individually.^44^ However, this approach places the responsibility on the patient, who often lacks sufficient information about PCa, and potentially overlooks the earlier onset of PCa observed among Black men that calls for screening at an earlier age. Given these conflicting and confusing recommendations, medical mistrust is believed to partly explain the significant health disparity in PCa among Black men.^22–24^

While Blacks are increasingly recognized as non-monolithic,^45^ in PCa research medical mistrust has only been researched among African Americans in an effort to understand its role in preventative health screenings and overall health outcomes.^25, 46, 47^ Indeed, few studies account for ethnic differences within the Black subgroups in the US when investigating PCa and/or medical mistrust^48–51^ and even fewer studies explore these issues using mixed methods to allow men’s perspectives to be more deeply explored. Such comprehensive studies are crucial to the development of culturally relevant, community-based interventions designed to reduce barriers to Black men’s preventative health screening uptake. Thus, this study seeks to address these crucial gaps in understanding.

The purpose of our mixed methods study was to assess how medical mistrust affects PCa screening behavior in ethnically diverse Black men and explore perceptions of medical mistrust in these subgroups.

## Methods

### STUDY POPULATION

Using community-based convenience sampling, Black men were recruited through local community groups, churches, word of mouth and in health fairs organized to provide free health screenings for Black men. Recruitment was conducted by health professionals who partnered with respective site leaders, holding townhall meetings to explain the study and distribute flyers. Inclusion criteria included men 21 years and older, English speaking, and who identified as African American (AA), African immigrants (AI) and Caribbean American (CA) immigrants. All participants consented prior to participating in the study, which was approved by Loma Linda University institutional review board (IRBs# 5110343 and 5190074).

### STUDY DESIGN

This was a mixed methods study including quantitative and qualitative phases. Community partner feedback revealed that to encourage participation from Black men in the study, holding health fairs where they can learn more about their health in general, would be beneficial. Thus, researchers, in collaboration with community partners, organized community health fairs specifically aimed to enegage Black men. Men who participated in these health fairs, underwent health testing (e.g., blood pressure checks, blood glucose measurements, BMI calculations), as well as screenings (e.g., cholesterol, rapid PSA tests). Each test was accompanied with intentional time to discuss the results and the reason for preventive monitoring as we aimed to promote preventive care and early detection of health issues. While awaiting the results of their PSA tests, participants completed surveys. Following the completion of the surveys and receipt of the PSA test results, medical doctors on-site provided counseling and education to the men regarding their PSA results and PCa. Due to the dependance on coordinating, planning and putting on health fairs, data collection occurred between January 2018 and March 2020.

Quantitatively, Black men (*n*=498) completed a survey assessing PCa screening history (ever screened for PCa) and intentionality (intention to screen for PCa), nativity (AA, AI, CA), and PCa family history (yes/no), in addition to medical mistrust. In this study, we assessed mistrust of healthcare organization and its impact on utilization of screening services because a patient’s interaction with the medical care system has become less focused on an individual physician and more focused with the system in which the health care delivery is taking place. A medical mistrust index^52^ was used and it asked respondents how they felt about the following statements: 1)You’d better be cautious when dealing with health care organizations, 2) Patients have sometimes been deceived or misled by health care organizations, 3)When health care organizations make mistakes they usually cover it up, 4)Health care organizations have sometimes done harmful experiments on patients without their knowledge, 5)Health care organizations don’t always keep your information totally private, 6)Sometimes I wonder if health care organizations really know what they are doing, and 7) Mistakes are common in health care organizations. Responses were scored from strongly disagree =1 to strongly agree =4. The medical mistrust scale had a Cronbach alpha of 0.83. Power calculations were conducted for binary outcome regressions, adjusting for demographic covariates. Within each subpopulation, there was 80% power to detect small effect sizes down to R^2^ = 0.095 with a sample size of 125 per subgroup and a total population size of n=375.

The qualitative phase included six focus groups with approximately eight individuals in each group (n=51) and ten key informant interviews (n=10). The qualitative data was collected at a later date than the health fairs (approximately 1 month after) with men who had attended the health fair and their partners who were aware of the health fairs. Interviews and focus groups were conducted in person and researchers used a semi-structured interview protocol (included as a supplemental). Interviews lasted 1 to 2.5 hours, were audio-recorded to ensure accurate transcription, transcribed verbatim and merged with field notes taken after the interview. Due to the verbatim transcription, transcripts were not given to participants for correction. We had no repeat interviews and no participants refused to participate. In the study, saturation was achieved after conducting nine out of ten interviews and the fourth out of six focus groups. At this point, no new themes or information surfaced during the data analysis, signifying that data collection had thoroughly addressed the pertinent topics.

Our study team received advice from our community partners to ensure gender concordance between interviewers and interviewees, as well as cultural alignment, due to the sensitive nature of the topic—PCa—and its connection to one’s sex life. Consequently, a female interviewer conducted interviews with women who had a male Black partner. We also interviewed clinicians, who were willing to discuss these topics with a female researcher. All other interviews and focus groups were conducted by male research assistants. All interviews and focus groups were conducted in English.

To maximize the credibility, dependability and confirmability of the findings,^53–55^ it was important to incorporate both forms of qualitative data collection (focus groups and key informant interviews) with our target population of varying ages, and the female partners of Black men. Using multiple methods and sources is a triangulation strategy that seeks validity through convergence in qualitative research increasing rigor,^56^ trustworthiness^53^ and comprehensiveness.^57^ Focus groups and interviews were conducted by trained qualitative, ethnically and gender matched interviewers at churches, community centers and participant’s homes. After analysis, data was disseminated at community meetings to share findings.

### REFLEXIVITY AND POSITIONALITY

The interviews were conducted by the first author and two additional research assistants. The interviewers were trained in qualitative methods in behavioral science and health services research and extensive prior experience interviewing and analyzing qualitative data. NM, the first author, is a female African immigrant who was a doctoral candidate during the data collection phase. She was supported by two male research assistants: one medical student identified as a Caribbean immigrant and the other identified as African American with a Master’s degree. Participants were informed of the interviewers’ ethnic backgrounds and the study’s objective of addressing the gap in PCa screening among Black men.

The remainder of the research team included a female registered nurse with a doctorate in public health, a female behavioral health researcher with a Ph.D., and two male Ph.D. researchers. All team members have a significant track record of engagement with Black communities nationwide, offering support and addressing health disparities. Before commencing the study, researchers collaborated with community leaders from each subgroup to establish rapport and brainstorm strategies for recruitment and dissemination of findings.

### STATISTICAL ANALYSIS

Quantitative data analysis was conducted using SAS version 9.4. Frequencies were generated and a logistic regression was used to explain the relationship between screening behavior and medical mistrust. Approximately 20% of data was missing at random and thus a multiple imputation method was used to deal with missingness.^58–60^

For qualitative data, all recordings were transcribed verbatim and assessed for accuracy by comparing recording and transcripts. A constant comparative method (grounded theory analysis) was used to analyze 16 different transcripts by two separate coders and disagreements were resolved using a third coder to assure transparency and rigor.^61^ Emergent line-by-line coding was completed and a final codebook was derived in a facilitated discussion to provided shared language for codes. The coders achieved an inter-rater reliability of 0.76, indicating an acceptable level of consistency in their assessment decisions. All transcripts were then coded using the final codebook and quotations that aligned with the codes were abstracted and clustered together to provide themes.

## Results

### QUANTITATIVE RESULTS

As presented in Table 1, 498 participants were surveyed; 231 self-identified as AA, 176 as CA and 91 as AI. The mean age was 49, 50 and 46 years, respectively. More CA reported having a family history of PCa, followed by AA and AI. Among AI, 36.7% had migrated to the US between the ages of 31 and 40 while 29% migrated between the ages of 21 and 30. In each subgroup, approximately 75% of participants had insurance. Among CA, the majority had migrated between the ages of 0 and 10 and 21% between the ages of 21 and 30. In each subgroup, individuals who reported ever undergoing a PSA screening comprised 58% AA, 56% CA, and 42% AI. Regarding DRE screening, 49% were AA, 45% were CA, and 36% were AI. Among those who reported having both PSA and DRE screenings, 39% were AA, 39% were CA, and 26% were AI.

Medical mistrust was highest among CA immigrants and statistically different between the three subgroups as determined by one-way ANOVA (F(2,557)=3.647, *p*=.02). A Tukey post hoc test revealed that there was no significant difference in mistrust between AI (17.54±4.21) and AA (17.82±4.23). AA reported lower medical mistrust than CA (18.81±3.93, *p*=.04). While CA had higher scores on medical mistrust than African immigrants, this was not statistically significant (*p*=.06). In a supplemental table, we explored factors that affected medical mistrust based on the literature, while controlling for the subgroups and everyday discrimination. Interestingly, being of CA descent showed a positive correlation with medical mistrust.

All results in Table 2 are reported holding all other variables in the model constant. Furthermore, all variables incorporated into the logistic regression analysis were chosen in accordance with the literature’s documentation of factors influencing screening behavior. In assessing previous screening behavior, for every one unit increase in medical mistrust, PSA screening increased by 11%. For one unit increase in age, having been screened increased by 13% for PSA, 8% for DRE and 9% for both PSA and DRE. For every one unit increase in income, past screening increased by 117% for PSA, and 87% for DRE and 120% for both tests. For every one unit increase in family history, previous DRE screening increased by 21%.

### QUALITATIVE RESULTS

The majority of the participants (76.4%) were 40 years and older and most were married (80.6%). Table 3 provides the descriptive characteristics of the qualitative phase. Since the quantitative results revealed differences in mistrust within the three subgroups and indicated that in our participants the impact of mistrust unexpectedly resulted in increased odds of PSA screening for PCa, we felt that it was important to further explore the issue of medical mistrust qualitatively. The results of the qualitative phase are presented below.

Overall five themes emerged: 1) root cause of mistrust, 2) medical mistrust, 3) cultural perceptions of seeking medical care, 4) negative past experiences with poor communication, and 5) the presence of PCa or knowledge of family history trumped mistrust. Most themes, however, varied somewhat across the three Black sub-groups, with men speaking of a lack of trust in the system, poor communication and lack of respect from their provideres. As a result, they preferred to avoid going to a physician in general and especially to talk about something as private and potentially impactful as PCa. However, all participants indicated that when PCa has a presence in one’s life (personal or family) it is important to stay on top of one’s risk.

#### Theme 1: Root cause of mistrust

Each subgroup described underlying reasons for mistrusting the medical system. For AA, 90% of respondents reported the historical precedence of Blacks not being treated fairly in the US. The Tuskegee history still rang true and more powerfully in today’s AA men and thus influenced their perceptions and behavior when it came to the medical system. Approximately 80% of CA also talked about the influence of history on mistrust but focused on the fact that Black people are never treated well by doctors or the medical system. Finally, for AI, 80% reported that medical mistrust of the medical system was rooted in a mistrust of Western medicine for it was seen as a byproduct of colonialism. Each of these subgroups presented with with mistrust, however, for each subgroup this mistrust stems from a different cause. AI, for instance, tend to link it less to personal experiences, which may be because they reported avoiding use of the system at all cost.

**Table:** 

**Root cause of mistrust**		

You know there was a study done in Tuskegee in which they inject black men with syphilis and the black men would go to the doctor and say this is what is going on and the doctor would say, oh you’re ok. And even though it was 40 black men, that permeated throughout the whole culture in the south and so the trust in the medical profession has been diminished because many times they see us not as patients but as a meal ticket. — African American	Black people and doctors and medicine have never had the best relationship. Look at our history, no matter where you are in the world, if you are a Black person, you will not be regarded as worthy, as someone worth treating with respect. — Caribbean Immigrant	You know, when they[the west/western medicine] brought condoms to African countries to tackle HIV, no one wanted to use them, not because they didn’t believe in prevention, but because people believed they[condoms] were coated with a virus. The West has never had the best interest for Africa and so when a vaccine comes out or a prevention, no one signs up because of our history with colonialism. And to make matters worse, they always send all their testing to Africa for vaccines and things like that, so are you surprised that no one want to trust such a system? — African Immigrant

#### Theme 2: Medical mistrust and cultural perceptions of seeking medical care

The perception of seeking medical care differs between AA and the two other Black immigrant subgroups and emphasizes the complex interplay between medical mistrust and cultural norms. For AA individuals, a fundamental lack of trust in the medical system significantly influences their decision-making process regarding seeking healthcare services. They perceive the medical system as profit-driven, viewing it with suspicion and skepticism. This sentiment is encapsulated by one participant’s assertion that medical professionals prioritize financial gain over patient well-being, thus fostering a reluctance to engage with healthcare services unless absolutely necessary. Consequently, AA individuals may opt to delay seeking medical attention or attempt to endure their symptoms independently.

Conversely, for Caribbean and African immigrants, cultural expectations play a pivotal role in shaping attitudes towards seeking medical care. Embedded within cultural norms is the perception that seeking medical care, particularly for men, signifies weakness. This belief fosters a stoic approach to health issues, wherein individuals are encouraged to endure symptoms rather than seek medical intervention. Participants from these two immigrant subgroups expressed a cultural aversion to seeking medical care unless absolutely essential, echoing sentiments of self-reliance and resilience.

In addition, gender and cultural concordance seem to be important to men as they influence their comfort levels, trust, and willingness to engage with healthcare providers, particularly in sensitive medical contexts such as PCa screenings. The majority (95%) of men preferred to have gender concordance as it related to PCa screening, and while cultural concordance was important, it was often discussed in terms of racial concordance. These perspectives shed light on the nuanced ways in which medical mistrust and cultural expectations intersect to influence healthcare-seeking behaviors among Black populations.

**Table:** 

**Medical mistrust and cultural perseptions of seekina medical care**		

If you have like diabetes they want, you to come see them on a regular basis and I hate to say that but I know that they use us like a money machine. They want us to come in for a test or a checkup but they do not try to cure us. As a man, you can take the pain, you can take the suffering so why go to the doctor for him to tell you that you are hurting? — African American I always went to a white doctor. I never went to a black doctor…When I went to the hospital they brought a [black] doctor to see me while in the hospital and the way she treated me changed…so I decided that I was going to stay with that doctor. When I go in there to see her it is so relaxed. When I go to see her she want to hear what’s wrong with you. — African American	I probably won’t go to the doctor until I am flat on my back. That is kind of the way I am. And I think though the culture as far as it concerns most Caribbean folk is that we don’t go to the doctor until we are flat on our backs. Until we can’t move. And I think it carries over to when we move to the states. Here we have access to very good medical care but we do not want to bother. When we feel something, we think we can fix it with some drink or something. We have to educate ourselves. — Caribbean Immigrant The first time I got checked for prostate cancer I was coming out of the Navy and it was a female doctor. She asked me if I am uncomfortable with her checking me for prostate cancer. I said, oh no doctor, I am certain you did this numerous times before. — Caribbean Immigrant	In Nigeria, you would hardly see a man who will carry himself to the hospital and say I am going for a check-up, it is very very rare and that same mentality can be carried here [U.S.] — African Immigrant It’s like you see your fellow black man and Ive had some these countless moments when somebody has come to the clinic and sees me (a clinician) and is happy because he has seen his brother. Brother from another mother taking care of him. — African Immigrant

#### Theme 3: Lack of education in relation to PCa

In conversations about prostate cancer (PCa) screening, most men (approximately 92%) from all three subgroups highlighted heightened mistrust stemming from a perceived lack of knowledge, since little or no education about the issues was shared by their providers. Moreover, they feel that doctors expect them to already possess knowledge about PCa, resulting in infrequent discussions about this during the already short and often busy appointments. In reality, Black men wanted and anticipate receiving education about PCa from their providers during their medical visits. Since this most often did not occur, this disconnect left men feeling hesitant to ask questions, further diminishing their trust in the medical system. Consequently, they seek information elsewhere, leading to uncertainty about its reliability.

**Table:** 

**Lack of Education in relation to PCa**		

For women when they have breast cancer for example they would tell women how to stand in front of a mirror and how to examine themselves and how to check themselves for breast cancer. For us we do not have that kind of Information. They do not tell us how to put your hands somewhere and do something. And I think for men, if we have education on how to exercise our prostate, we wouldn’t have these problems. But my doctor has never talked to me about it — African American People forget that in America the institution of slavery kept a lot of these things…we couldn’t educate ourselves. We weren’t able to learn; we weren’t able to read. It wasn’t until 1954 when Dred Scott, when the decision was made for us to be able to go to school again after segregation that we could actually learn. there were barriers put in place beyond our control. We have just gotten out of that now and now we are supposed to know how to take care of ourselves? Who is going to tell us? Who is going to teach us? — African American	You only go see a doctor once or twice a year and they only see you for a few of minutes. Meanwhile there is a lot of stuff going on that you need to talk to him about but the time is not there. So, I use the internet but there is a whole set of stuff that comes up about prostate cancer and men’s health that you can’t even know what’s true and what’s false — Caribbean Immigrant	I am ready to acknowledge that I don’t know anything that directly contributes to prostate cancer. I don’t know what I should be doing to prevent prostate cancer and my doctor hasn’t shared that with me. I think if you live long enough you are probably going to develop some level of prostate cancer. So, I am not going to even worry about it because by the time I get it, if I get it, I am almost dead anyway. — African Immigrant I was talking with someone who said that diet and some of these things are good to be attentive to. He also discussed sexuality and talked about the different approaches to sex and the timing and those things. Something about the frequency of sex associated with better prostate health. He in fact observed that he and his wife are applying these finding to their lives. I want to believe this is true but I just don’t know — African Immigrant

#### Theme 4: Negative past experiences and poor communication

Irrespective of their subgroup, men expressed that their previous and recent negative encounters with healthcare professionals or the medical system have amplified their mistrust. Many men recounted past experiences that left them apprehensive about future interactions with doctors. They perceived physicians as distant, unapproachable, and critical, often recalling instances where they felt belittled and unfairly judged. Instead of fostering engagement, physicians often proceed with procedures without adequately communicating or explaining them, leaving men feeling disregarded. Furthermore, when issues arise, there’s a lack of acknowledgment or apology for the stress caused. Men also noted inconsistencies in information provided by different healthcare professionals and felt frustrated by a lack of effort to explain potential complexities, instead being treated in a patronizing manner, as though they should unquestioningly comply.

**Table:** 

**Negative Past Experiences and poor communication**		

I went to a doctor once with blood pressure high, well…it was always high but when I went to the doctor it was higher because something was always coming up. You are going to die, you know. He was stacking me up on medication. Nothing that I said, he really wanted to hear. He just said, well you are not doing so and so. And so, I really did not want to go to him. I did not want to go to him because he would say you are too fat, you’re too this, you’re too that. — African American Now that I know that I have it[prostate cancer] you know…how to get rid of it did not come to my mind. I went to the doctor and talked to him again and he told me what my Gleason index was and stuff like that. I went online and checked on the things that he told me and the meaning of the scores. Then they referred me to a specialist who deals with that. Then I said, now what is my path to follow in dealing with it? What do I have to do to deal with it because the doctor just dealt with me for a few minutes and then he told me of the months in between that I have to deal with it. So, those were hard times. — African American	It was a Korean doctor; I knew he worked there. When he was coming to the end he said, bend over. He just said pull down your pants and bend over. And he was so rough, he pushed up his finger. After he was finished I sat down and I cried. It hurt. It hurt. He gave no explanation nor said what he was doing — Caribbean Immigrant My urologist who was my expected surgeon indicated that I had a 75% to 85% success rate of sparing the erectile functioning nerve and that was important because I am in my early 40s. and so when I went into the place for pre-admission and the nurse practitioner is telling me that I need to sign the documents and there is a 50/50 chance, because if we go in and anything causes a complication, they will need to do a radical prostatectomy. This would lead to possibility of impotence because of surgery. I left there confused. I was frustrated. I was angry. I was here wondering, here the doctor told me about 75–80% success to avoid impotence and now you are telling me about 50/50. And I was signing up for surgery which was just around the corner. I was mad with them. — Caribbean Immigrant	I went for a physical once and the doctor took my blood and ran some other tests and next thing they tell me is that my white blood cell count was low, I might have HIV and that my cholesterol is way too high. I couldn’t sleep for a week. I was stressing out every day and then they called me and said, ‘oh we made a mistake. We switched your results with someone else.’ You have got to be kidding me. Do you realize how wrong that is and how biased it is to assume that just because I am from Africa and my white blood cell count may be low, that I might have HIV. That’s just wrong. — African Immigrant I think they do a lot of trial and error tests on people. Based on how I was raised, I will try home remedies before going to the doctor. I try a lot of preventive measures and when an issue arises, I will weigh the situation and the urgency because doctors means money. And uncertainty. And doctors sometimes don’t talk you through what they are doing, why they are doing it and how much it will cost. — African Immigrant

#### Theme 5: Presence of PCa or knowledge of family history trumped mistrust

While reports and reasons for mistrust towards doctors and healthcare organizations may have influenced men’s preventive actions, the presence of a family history (knowing someone with PCa, witnessing someone’s death from PCa, or having a personal diagnosis of PCa) by itself, tends to encourage them to engage with the healthcare system. This seemingly paradoxical behavior arises from their desire to prolong their lives or avoid experiencing what they witnessed in a family member’s struggle with PCa. This did not change their feelings of mistrust. Indeed, each subgroup acknowledged the validity of their mistrust and views their reluctance to engage with the healthcare system as justified. However, upon receiving a diagnosis of PCa and understanding its severity, their behavior underwent a significant change as now a survival response was needed. Indeed, a PCa diagnosis overrode their mistrust and past negative experiences, compelling them to ignore these, enagege with the health care systems, and advocate for their own health despite their initial hesitations.

**Table:** 

**Presence of PCa or knowledge of family history trumped mistrust**		

I have an uncle who died from prostate cancer. He was 96 and he was a vegetarian. He did not go and get checked. You know, as a matter of fact, he felt something and he did not do anything about it. That’s just crazy, I will go — African American	My father had it. It really affected me because I took care of my dad. I watched him die. I understand how devastating that cancer was. I don’t care how much they[healthcare organization/personnel] are against us, if there is any hope for me to not have the same fate as my dad, I will go. — Caribbean Immigrant Personally, when my father had it, it affected him. He fit the stereotype…strong, Caribbean American male. When he found out it was in his later years of life… Surgery option? No. he did not want to have surgery. Afraid of the knife [saying] I am not going to wake up, but at the same time he knew he had no other option. When you are facing death, your perceptions and opinions go out the window and you just go and trust they will do right by you — Caribbean Immiarant	My cancer is the lowest stage possible. In fact, my treatment now is a wait and see type of thing in which they monitor what I eat and so on it is called active surveillance. It doesn’t matter what I think about the system, I have it, so I to keep going in — African Immigrant

## Discussion

To the best of our knowledge, this study represents the first attempt to evaluate potential differences regarding the impact of medical mistrust on PCa screening behavior among ethnically diverse Black men, as well as to delve into their perceptions of medical mistrust within these subgroups. This is crucial because mistrust of healthcare professionals and organizations has been associated with less treatment adherence, less utilization of healthcare services and less care satisfaction, which may be a contributing factor to the disparity Black men have in PCa.^23, 62, 63^

Researchers have reported higher levels of medical mistrust by AA in comparison to other racial groups.^64, 65^ However, when we disaggregated our data to Black ethnic sub-groups, this study revealed that although AA reported a high rate of medical mistrust of healthcare organizations, Caribbean Black men report higher rates of mistrust than both AA and AI men. This is an important finding because it further supports recent findings that American Blacks are not a monolithic group, but rather, differences exist between the subgroups and that this holds for the issue of medical mistrust.^45, 50, 51^ Further investigation on factors that affect mistrust were quantitatively assessed and everyday discrimination was significantly correlated with medical mistrust. This is in alignment with literature and suggests that perceived discrimination in one’s life does influence medical mistrust; thus, to address one facet of mistrust, one must address discrimination.^66^

Not surprisingly, age and income were identified as significant factors influencing screening for either PSA, DRE, or both. Notably, increased income exerted a more substantial impact on screening behavior compared to other factors. This finding is consistent with existing literature, which indicates that individuals with higher incomes tend to avail themselves of preventive health services, including cancer screening and immunizations, at higher rates due to enhanced accessibility.^67^ Additionally, it’s worth noting that approximately three-quarters of each subpopulation had medical insurance, suggesting a likely correlation between income and insurance coverage. These findings align with existing literature^68, 69^ and emphasize the importance of addressing socioeconomic disparities to ensure equitable access to essential healthcare services, thereby advancing public health initiatives aimed at reducing the burden of PCa.

Our findings also revealed that despite harboring medical mistrust, participants remained willing to undergo PSA screening, albeit not for DRE or both PSA and DRE. This observation is in alignment with prevailing literature suggesting that medical mistrust reduces preventive health screening rates.^23, 70^ Recognizing the significance of this result, we sought to conduct a deeper qualitative exploration. Our qualitative analysis unveiled that reluctance to have a DRE was related to provider communication around this issue, which was seen as quite poor and they felt taken aback and offended as this test was conducted without prior discussion. Additionally, our previous work^51^ also shows that Black men(AA, CA, and AI) are apprehensive about undergoing more invasive PCa screening, such as the DRE, due to the perceived instrusivness of the procedure and fears around losing their masculinity if they were to undergo such a procedure. Moreover, beyond seeking DRE, Black men consciously chose to prioritize the seriousness of PCa over their general reluctance to engage with the healthcare system, despite perceiving bias against the Black community within the system. Notably, this perspective was primarily shared by individuals who had a close family member or friend with PCa or had personally experienced a PCa diagnosis. It appears that when confronted with the reality of PCa in their lives, their priorities regarding this specific preventive behavior shifted. Our qualitative findings were consistent with this notion, as men expressed the challenge of setting aside their usual reluctance to engage with the healthcare system when confronting PCa. They shared how difficult it was to deviate from their typical perspective but recognized it as necessary. Participants discussed feeling compelled to take control of their lives, driven in part by their mistrust of the system and the need to monitor their health closely. Witnessing the impact of PCa or experiencing it firsthand motivated them to overcome past negative experiences and actively engage with the healthcare system to address the risks associated with PCa.

Expanding on this discovery, our investigation into potential differences in medical mistrust among the subgroups revealed that while all exhibited high levels of distrust, the underlying reasons varied. AA predominantly attributed their mistrust of the healthcare system to historical precedents, a finding extensively supported in literature associating Black mistrust with events like the Tuskegee Syphilis study and other historical mistreatments.^24, 71^ Caribbean immigrants, boasting the highest levels of medical mistrust among all subgroups, acknowledged the significant influence of history on their mistrust. However, they also emphasized their personal experiences, noting that they and other Black individuals are generally poorly treated by doctors or the medical system. For AI, medical mistrust stemmed from a distrust of Western medicine, perceived as a legacy of colonialism where Black individuals are marginalized or provided with subpar care options. Additionally, both CA and AI argued that their reluctance to visit the doctor was influenced by cultural perceptions that seeking medical help is a sign of weakness for males. This reluctance was further supported by their shared experiences of not feeling heard, respected, or included in medical decision-making, which led to higher levels of medical distrust. Ironically, this distrust translated to higher levels of PCa screening for some, as the three Black subgroups felt they had to look out for themselves since they believed the medical care system would not do so. For them, it was all about survival, especially once they learned about their higher risk for PCa and witnessed loved ones suffering from the illness, often at late stages due to a lack of early diagnoses. This cultural perspective – a strong male identity expectation combined with a healthcare system that did not care about them – aligns with existing literature, which highlights arguments around masculinity and cultural beliefs as factors contributing to men’s underutilization of health services.^8, 10, 15^ In Caribbean and African cultures, visiting the doctor is often seen as a last resort, as it may be interpreted as a demonstration of weakness.

Furthermore, men also attributed their mistrust to a lack of knowledge and unmet educational expectations from their healthcare providers. Our participants demonstrated awareness of their need for more information and hoped to receive it from their providers. However, they reported that this expectation often went unfulfilled, as providers rarely communicated with them or took the time to explain things thoroughly. Additionally, we observed that many men were aware of significant misinformation among health care providers about when to screen, particularly for Black males. This likely stemmed from a lack of clear guidance among health care organizations setting policies. This lack of guidance led to providers failing to clearly communicate the necessity ad the risks associated with PCa treatment, acting as a deterrent for these men. Many recounted experiences where physicians conducted physical exams without providing adequate explanations, and if results were unfavorable, there was a lack of further discussion or provision of resources. This issue was reported universally among Black men, regardless of their ethnic subgroup. Understanding this is critical because addressing the high rates of morbidity and mortality from PCa among Black men necessitates timely preventive screening and, if necessary, a comprehensive explanation of treatment options.

### Limitations

There are several important limitations to our study that require acknowledgment. First, in our qualitative phase, medical mistrust was examined alongside other aspects of PCa screening, such as knowledge, past experiences, and other barriers. Consequently, it is possible that we may not have captured all facets of medical mistrust. Secondly, our quantitative phase employed a cross-sectional design, which restricts our ability to establish causal relationships. Additionally, we relied on self-reported data, raising the possibility that participants may have responded in a socially desirable manner. It’s pertinent to note that our examination solely centered on ever screening behavior rather than recent screening behavior, predominantly due to the scarcity of individuals who had undergone screening within the past year among Black men. The limited number of participants who had screened in the last year rendered it impractical to include this variable in our statistical analysis. Despite these limitations, the inclusion of data from various states across the US, which provided access to subgroups of Black men (including participants from most Caribbean islands and north, east, west, and central Africa), is a notable strength of our study. Furthermore, our success in recruiting large numbers of Black men through diverse recruitment sites such as local churches, community centers, and organizational groups, underscores the strength of our approach and has broader implications for the design of future interventions.

## Conclusion

This study uniquely contributes to existing health disparities research by exploring how PCa screening behaviors are impacted by medical mistrust in AA, CA, and AI Black subgroups. Our findings underscore two key summative points: 1) while all Black men may share some similar opinions and negative perceptions and experiences with the healthcare system, there are differences within subgroups in regards to medical mistrust rates and reasoning; and 2) medical mistrust may not be a critical deterrent of healthcare utilization among those with a family history or diagnosis of PCa, which in turn provides an opening for future interventions to engage these groups of men who continue to experience stubbornly high, disproportional rates of PCa.

## Supplementary Material

Supplemental Interview Guide

## Figures and Tables

**Table 1.** Demographic Characteristics (n=498)

	African Americans	Caribbean Immigrants	African Immigrants
	n=231	n=176	n=91

Age M(SD)	49.17 (15.01)	50.26 (14.44)	46.72 (12.78)
Marital Status			
Single (never married)	73 (31.60)	47 (26.70)	16 (17.58)
Married (first marriage)	138 (59.74)	125 (71.02)	63 (69.23)
Re-married	14 (6.06)	2 (1.14)	6 (6.59)
Separated/Divorced	3 (1.30)	2 (1.14)	6 (6.59)
Widowed	3 (1.30)		
Income			
Low income (< $44,999)	104 (41.60)	86 (46.49)	27 (32.14)
Middle class ($45,000–125,000)	107 (42.80)	77 (41.62)	44 (52.38)
High income class (> $125,001)	39 (15.60)	22 (11.89)	13 (15.48)
Family Members with PCa			
0	35 (48.61)	9 (15.79)	51 (77.27)
1 or more	37 (51.39)	48 (84.21)	15 (22.73)
Medical Insurance			
Yes	197 (72.96)	146 (71.92)	72 (75.00)
No	73 (27.04)	57 (28.08)	24 (25.00)
Medical Mistrust M(SD)			
Yes	197 (72.96)	146 (71.92)	72 (75.00)
No	73 (27.04)	57 (28.08)	24 (25.00)
Medical Mistrust M(SD)			
Low Mistrust	51 (19.5)	26 (12.7)	21 (22.3)
High Mistrust	211 (80.5)	179 (87.3)	73 (77.7)
Past PSA Screening	90 (58.4)	71 (56.8)	21 (42.0)
Past DRE Screening	76 (49.3)	59(45.5)	18 (36.7)
Past PSA and DRE Screening	60 (39.2)	49 (39.5)	13 (26.5)
Age of Migration			
0–10yrs		69 (42.9)	9 (13.2)
11–20yrs		5 (3.1)	6 (8.8)
21–30yrs		35 (21.7)	20 (29.4)
31–40yrs		31 (19.3)	25 (36.7)
41–50yrs		15 (9.3)	5 (7.5)
>51yrs		6 (3.7)	3 (4.4)

**Table 2.** Factors that have affected past screening behavior

	Past PSA Screening		Past DRE Screening		Past Screening for Both	
		
	*OR*	*CI*	*OR*	*CI*	*OR*	*CI*

Age	1.13***	1.08,1.17	1.08***	1.05,1.13	1.09***	1.05,1.13
Black subgroup						
African Americans	1.00	Ref	1.00	Ref	1.00	Ref
African immigrants	0.54*	0.21,1.44	0.46*	0.19,1.14	0.50	0.18,1.35
Caribbean immigrants	1.70	0.58,4.95	1.03	0.40,2.65	1.16	0.43,3.13
Income	2.17**	1.19,3.95	1.87*	1.09,3.23	2.20**	1.23,3.94
Family History	1.59	0.84,3.03	1.21*	0.73,2.00	1.57	0.93,2.66
Medical Mistrust	1.11*	0.99,1.23	0.97	0.88,1.07	0.97	0.88,1.07

* p<0.05

** p<0.01

*** p<0.001

**Table 3.** Qualitative Demographics

Characteristics	African Americans		Caribbean immigrants		African Immigrants	
		
	Men (*n*=14)	Women (*n*=7)	Men (*n*=14)	Women (*n*=9)	Men (*n*=13)	Women (*n*=5)
		
	n(%)	n(%)	n(%)	n(%)	n(%)	n(%)

Education						
High School graduate	3 (21.4)	0 (0.0)	2 (14.3)	0 (0.0)	0 (0.0)	0 (0.0)
Some College	4 (28.5)	2 (28.5)	3 (21.4)	1 (11.1)	0 (0.0)	0 (0.0)
Bachelor’s Degree	4 (28.5)	5 (71.4)	4 (28.5)	5 (55.6)	5 (38.5)	2 (40.0)
Master’s or Professional Degree	3 (21.4)	0 (0)	5 (35.7)	3 (33.3)	8 (61.5)	3 (60.0)
Income						
Low income (< $44,999)	6 (42.8)	1 (14.3)	3 (21.4)	3 (33.3)	3 (23.1)	0 (0.0)
Middle class ($45,000–125,000)	6 (42.8)	6 (85.7)	4 (28.5)	4 (44.4)	8 (61.5)	5 (100.0)
High income class (< $125,001)	2 (14.3)	0 (0)	7 (50.0)	2 (22.2)	2 (15.3)	0 (0.0)
Insurance						
Yes	11 (78.5)	5 (71.4)	12 (85.7)	8 (88.9)	11 (84.6)	5 (100)
No	3 (21.4)	2 (28.5)	2 (14.3)	1 (11.1)	2 (15.4)	0 (0.0)
