# Supplementary material for: Medical Mistrust on Prostate Cancer Screening: A mixed method study among African Americans, Caribbean immigrants and African immigrants
Source: Med Res Arch. Author manuscript; Available in PMC 2024 Oct 10. (PMC11466240; doi:10.18103/mra.v12i8.5727)
Supplement: Supplemental Interview Guide [file NIHMS2021384-supplement-Supplemental_Interview_Guide.docx]

**Supplemental Interview/Focus Group Guide**

**Ice-breaker Questions**

1. If you could choose 3 adjectives to describe yourself, what words would you choose?

**Main Questions**

1. What does “health” mean to you as a male and in particular as an African male (pattern of a male)? Give us some examples.
   1. To your partner/ your family
   2. How does stress fit?
2. What are some of the “highs” and the “lows” of your lives?
   1. Relationships
   2. Separation from country
   3. Role as a male in society
3. What are some of the issues that are important to you in terms of men’s health?
   1. Prostate cancer- what do you know about it?
   2. Do you ever talk about it? – With your partner, other men like you?
   3. Do you know anyone who has had or has prostate cancer- who – what were their experiences?
4. What role do you play in the maintenance of your health?
   1. Particularly in prostate health issues?
   2. General health screening issues?
5. How about prostate cancer screening?
   1. Have you considered getting such testing done? Why /why not?
   2. Is it important to your family? Was it discussed in the family? Was it discussed with any other men like you? Did you discuss details about it i.e. digital rectal examinations and prostate specific antigen tests (PSA tests); why and why not?
   3. Have you ever discussed these issues with your doctor? Did you think they were important enough to discuss them with your doctor?
   4. What are the positive sides and what the negative sides of testing (benefits)?
   5. How about if you found out you have cancer—what would you do?
6. Can you describe for me how men talk about prostate cancer?
   1. Do men think there are things they can do to prevent getting prostate cancer
   2. What should one do to reduce risk of prostate cancer
   3. How about stress, diet, exercise? Tell me a little about those things.
   4. What do men fear the most when they think about prostate cancer?
7. Tell me what you heard how men may deal with a diagnosis of prostate cancer?
8. Tell me about how your family may deal with a diagnosis of prostate cancer? Tell me about how you talk about it…tell me about how it may affect your relationship…is it possible that you got/may get closer as a result of the diagnosis?
9. How might this affect the way men feel about their own manhood or masculinity?
10. Tell me how so?
11. How about sex/closeness?
12. Do you feel that beliefs men hold about life and health in general affected the way you deal with issues like prostate cancer screening and the diagnosis of prostate cancer?
    1. Tell me more about your beliefs. Do you think that some things must happen a certain way no matter what? Do you think there is nothing a person can do to avoid certain things? Some people call that fatalism or fate, do you believe in that? Tell me some more.
    2. How about God/ a higher power?
    3. Fate? Personal Responsibility
13. Tell me a little about your family communication. Do you discuss things in general a lot?
    1. Do you talk about how you look at health/illness?
    2. Do you discuss health care decisions as a family/couple?
    3. Who would be the first person you would consider sharing a diagnosis of prostate cancer with? – How soon?
    4. Do you discuss prostate cancer screening decisions as a couple/family?
14. What may/does having a diagnosis of prostate cancer mean to people in general?
    1. Men?
    2. To you an individual
    3. To you as a couple?
    4. How should we educate black men about health in general?
    5. How about stress
    6. How about prostate cancer and the benefits of early detection?
15. Where do you think men get their beliefs about prostate cancer from?

**Exit Questions**

1. Of all the things that we discussed today as they relate to you, your family relationship, prostate cancer screening behaviors, prostate cancer diagnosis, fatalism, and spirituality, what would you say is the most important?
2. If you had all the resources you needed and could help men with this issue (prostate cancer—how would you go about helping other men with this?
